# Altered Intracortical T_1_-Weighted/T_2_-Weighted Ratio Signal in Huntington’s Disease

**DOI:** 10.3389/fnins.2018.00805

**Published:** 2018-11-05

**Authors:** Christopher D. Rowley, Sarah J. Tabrizi, Rachael I. Scahill, Blair R. Leavitt, Raymund A. C. Roos, Alexandra Durr, Nicholas A. Bock

**Affiliations:** ^1^McMaster Integrative Neuroscience Discovery and Study Program, McMaster University, Hamilton, ON, Canada; ^2^Huntington’s Disease Centre, University College London Institute of Neurology, National Hospital for Neurology and Neurosurgery, London, United Kingdom; ^3^Department of Medical Genetics, The University of British Columbia, Vancouver, BC, Canada; ^4^Department of Neurology, Leiden University Medical Center, Leiden, Netherlands; ^5^INSERM U1127, CNRS UMR7225, UMR_S1127, UPMC Université Paris VI, Institut du Cerveau et de la Moelle Epinière, Sorbonne University, Paris, France; ^6^APHP, Department of Genetics, Pitié-Salpêtrière University Hospital, Paris, France; ^7^Department of Psychology, Neuroscience and Behaviour, McMaster University, Hamilton, ON, Canada

**Keywords:** Huntington’s disease, MRI, cerebral cortex, myelin, neurodegeneration

## Abstract

Huntington’s disease (HD) is a genetic neurodegenerative disorder that is characterized by neuronal cell death. Although medium spiny neurons in the striatum are predominantly affected, other brain regions including the cerebral cortex also degenerate. Previous structural imaging studies have reported decreases in cortical thickness in HD. Here we aimed to further investigate changes in cortical tissue composition *in vivo* in HD using standard clinical T_1_-weighted (T_1_W) and T_2_-weighted (T_2_W) magnetic resonance images (MRIs). 326 subjects from the TRACK-HD dataset representing healthy controls and four stages of HD progression were analyzed. The intracortical T_1_W/T_2_W intensity was sampled in the middle depth of the cortex over 82 regions across the cortex. While these previously collected images were not optimized for intracortical analysis, we found a significant increase in T_1_W/T_2_W intensity (*p* < 0.05 Bonferroni-Holm corrected) beginning with HD diagnosis. Increases in ratio intensity were found in the insula, which then spread to ventrolateral frontal cortex, superior temporal gyrus, medial temporal gyral pole, and cuneus with progression into the most advanced HD group studied. Mirroring past histological reports, this increase in the ratio image intensity may reflect disease-related increases in myelin and/or iron in the cortex. These findings suggest that future imaging studies are warranted with imaging optimized to more sensitively and specifically assess which features of cortical tissue composition are abnormal in HD to better characterize disease progression.

## Introduction

Huntington’s disease (HD) is an autosomal dominant neurodegenerative disorder that is caused by a CAG repeat expansion in the *HTT* gene (MacDonald et al., 1993). This repeat expansion leads to disrupted signaling in cortico-striatal circuits, followed by a loss of projection neurons in the striatum (Rangel-Barajas and Rebec, 2016). The neuronal loss and disrupted communication within the brain lead to the characteristic traits of the disease: progressive motor dysfunction, cognitive decline, and behavioral deterioration (Walker, 2007; Ross and Tabrizi, 2011). Given that symptomology extends beyond basic motor dysfunction into mood and cognition, the cerebral cortex may be implicated in the pathology of HD. In fact, postmortem studies have suggested that neuronal losses are prevalent in the cortex (Hedreen et al., 1991; Selemon et al., 2003; Kim et al., 2014; Mehrabi et al., 2016) which warrants *in vivo* investigations in patients.

Since HD is progressive, *in vivo* imaging methods are invaluable for identifying disturbances in brain structure or function over the course of the disease. MRI specifically has shown alterations in the brains of HD patients. These findings have primarily been structural in nature, with decreases in volume reported in the striatum, thalamus, cortical GM, and major subcortical WM tracts (Aylward et al., 1998; Thieben et al., 2002; Rosas et al., 2003; Fennema-Notestine et al., 2004; Tabrizi et al., 2009, 2011, 2012; Paulsen et al., 2010; Stoffers et al., 2010). Previous reports of cortical thinning in HD suggest that thinning begins in motor and visual regions of the cortex and spreads to nearby cortices with HD advancement (Rosas et al., 2002, 2008; Tabrizi et al., 2009). In terms of cerebral tissue composition, MRI has revealed increases in iron levels in the striatum in HD (Bartzokis et al., 1999, 2007) and most recently, diffusion MRI and quantitative magnetization imaging have suggested a breakdown of myelin in deep WM tracts in HD (Bourbon-Teles et al., 2017). Additionally, diffusion tensor imaging (DTI) and functional MRI (fMRI) have been used to display changes in the HD brain. DTI has highlighted that microstructural changes in WM increase with HD progression (Rosas et al., 2006; Weaver et al., 2009), that WM microstructure scores are correlated with motor measures (Dumas et al., 2012a; Poudel et al., 2014, 2015), and that aberrant network connectivity develops in the HD brain (Poudel et al., 2014). A compensatory mechanism in the presymptomatic HD brain has been revealed with fMRI studies, where several cortical and subcortical areas have shown increased event-related activation (Paulsen et al., 2004; Klöppel et al., 2015). However, it was shown that cortical activation starts to decrease in symptomatic HD patients (Domínguez et al., 2017). Taken together, MRI provides a sensitive tool for investigating the progressive degeneration of the HD brain.

Although the cortical thickness studies suggest cortical involvement in HD, cortical thickness is a broad measure of cortical pathology that is sensitive to a range of microstructural features (Zatorre et al., 2012). This study aimed to investigate HD progression in the cerebral cortex using intracortical T_1_W and T_2_W MR signal intensity as a potential marker of changes in cortical tissue composition. In the brain, T_1_ (Stüber et al., 2014) and T_2_ (Desmond et al., 2016) are sensitive to myelin and iron amounts and can be used to measure tissue water content (Neeb et al., 2006). Previously, the T_1_W/T_2_W ratio has been used to study the relationship between intracortical myelin levels and aging (Grydeland et al., 2013; Shafee et al., 2015), the trait of openness (Yasuno et al., 2017), performance stability (Grydeland et al., 2013), and error processing (Grydeland et al., 2016). The ratio of T_1_W and T_2_W images has a reduced sensitivity to bias (Glasser and Van Essen, 2011), aiding in removal of much of the bias artifacts arising from a multi-site study, which may obscure subtle disease-related changes. While these ratio maps have been suggested to map myelin across the cortex (Glasser and Van Essen, 2011), they might not be entirely specific to myelin (Arshad et al., 2017), especially in the presence of iron or cellular density changes that may be present in HD. Thus, in the present study we interpret the ratio image intensity as a general marker of cortical composition changes in HD rather than being specific for myelin changes.

This study utilizes previously collected MRIs from the cross-sectional TRACK-HD study (Tabrizi et al., 2009), which contains a large number of well-described subjects that were imaged at high resolution with multiple MR contrasts including the requisite T_1_W and T_2_W for our analysis. The dataset represents disease stages across HD, starting before disease onset to well after the disease has progressed, which permits the characterization of cortical changes with HD advancement. The neuroimaging portion of the TRACK-HD study (Tabrizi et al., 2009) was optimized for gray/white matter contrast to investigate gross structural brain changes. In doing so, they previously revealed widespread decreases in thickness and volume of cortical GM in HD brains. Thus, this study examines the utility of mapping intracortical MRI signal using standard clinical T_1_W and T_2_W images to further characterize HD.

## Materials and Methods

TRACK-HD imaging and participant recruitment information has been detailed previously (Tabrizi et al., 2009). The main points are summarized below.

### Participants

Three hundred and sixty six subjects were recruited from four imaging centers. Each center aimed to recruit a sample of 90 subjects with the following composition: 30 controls, 30 participants with premanifest HD, and 30 participants with early HD. There were 366 images made available to us, with the following distribution: 123 controls, 120 premanifest HD, and 123 early HD.

Premanifest subjects were excluded with a burden of pathology score lower than 250, and had a total motor score below 5 using the motor assessment of the United Huntington’s Disease Rating Scale (UHDRS). Healthy control subjects were age- and gender-matched to the combined HD and preHD group. To help control for environmental factors, controls were chosen from spouses or partners of preHD subjects, or were gene-negative siblings. This strict selection criterion aids in associating reported changes with HD.

Huntington’s disease participants were further subdivided for analysis. Premanifest HD subjects were split based on the median time for the predicted years to diagnosis (Langbehn et al., 2004) into those far from onset (PreHD-A) and closer to onset (PreHD-B). Early HD subjects were split based on their score on a total functional capacity scale (TFC) (Shoulson and Fahn, 1979) into Stage 1 (HD1, TFC 11–13) and Stage 2 (HD2, TFC 7–10).

In total 40 subjects had data that was not technically suitable for analysis: 19 subjects were excluded in primary stages of processing due to inadequate intrasubject image registration and 21 subjects were excluded in the analysis due to low signal-to-noise ratios in the MRIs, or obvious gain differences between the T_1_W and T_2_W images. Subjects were removed if their mean cortical intensity was outside mean ± 1.5× the standard deviation of the entire cohort, which would suggest a gain difference between the T_1_W and T_2_W images. The remaining 326 subjects used in the study are summarized in Table 1.

**Table 1:** Demographics for the participants used from the baseline TRACK-HD dataset.

	Controls (*n* = 112)	PreHD			HD		
		PreHD-A (*n* = 54)	PreHD-B (*n* = 51)	Combined (*n* = 105)	HD Stage 1 (*n* = 68)	HD Stage 2 (*n* = 41)	Combined (*n* = 109)
Age (years)	46.1 (10.5)	40.6 (8.7)	39.6 (8.8)	40.1 (8.9)	47.1 (10.2)	50.9 (8.8)	48.6 (9.8)
Women	60 (54%)	30 (56%)	29 (57%)	59 (56%)	40 (59%)	19 (46%)	59 (54%)
Disease-Burden score	N/A	259.8 (29.0)	335.4 (30.2)	296.6 (48.2)	357.8 (74.5)	397.2 (69.7)	372.7 (75.3)
**Centers**							
Site #1	25	14	11	25	15	12	27
Site #2	29	14	16	30	19	11	30
Site #3	29	13	15	28	23	4	27
Site #4	29	13	9	21	11	14	25

*Disease-burden score = Age × (CAG length – 35.5). Data values are mean (SD), or number (%).*

### Imaging

All images were collected on 3T whole body scanners from two vendors (Siemens [S] and Phillips [P]). T_1_W images were collected using a 3D MPRAGE acquisition with the following parameters: TR = 2200 ms [S]/7.7 ms [P], TE = 2.2ms [S]/3.5 ms [P], TI = 900ms [S]/950 ms [P], FA = 10° [S]/8° [P], FOV = 28 cm [S]/24 cm [P], matrix size 256 × 256 [S]/224 × 224 [P], 208 [S]/164[P] sagittal slices with 1 mm thickness to cover the entire brain. T_2_W images were acquired (SPACE sequence on Siemens scanners, VISTA on Phillips scanners) with identical field of view, acquisition matrix, slice thickness and the following timing parameters: TR = 3000 ms, TE = 421 ms.

### Processing

The aim of the image processing was to accurately segment GM and WM tissue classes in each subject’s T_1_W MRI and construct a middle-depth surface through the cortex for visualizing intracortical MRI signals. The middle depth surface of each subject was registered to a template to allow for group comparisons using a ROI analysis (Supplementary Table S1).

The software used for image processing was: MATLAB (vR2015a^1^), Elastix (Klein et al., 2010; Shamonin et al., 2013), ITK-SNAP (version 3.4^2^) (Yushkevich et al., 2006), MIPAV v7.4 software^3^ using the JIST v3.0 ^4^, TOADS-CRUISE vR3c ^5^, and CBS High-Res Brain Processing Tools Version v3.0 ^6^ plug-ins.

First, images were rigidly registered to begin the cortical segmentation. Each subject’s T_2_W image was rigidly registered to that subject’s T_1_W image using Elastix. The T_1_W image was rigidly registered to the 1 mm ICBM-152 asymmetric template^7^, and then the 6-parameter affine transform was applied to the T_2_W image such that both images were co-registered in ICBM-152 space.

Cerebral tissue segmentation was performed on the T_1_W image using custom scripts in MATLAB. First a custom written algorithm was applied to perform local intensity normalization to the T_1_W image to aid in segmentation of the pial surface where cortical signal is artificially reduced in some images due to bias in radiofrequency transmit and receive fields (B_1_+ and B_1_-). Briefly, a new image was created by sliding a 3 × 3× 3 window across the original T_1_W image, with the center voxel location in the new image taking on the greatest intensity value in the original image. This image was smoothed using a 10 mm 3D Gaussian kernel creating an intensity field map. The original T_1_W image was divided by this new image, creating an image where intensity is normalized by nearby voxels. This image was used solely for image segmentation and not for subsequent MRI signal analysis, since meaningful changes in intracortical signal are also potentially normalized.

Next the ICBM-152 template was registered to the subject using a b-spline transform, and the transformation was applied to a smoothed brain mask that had been created in ICBM-152 space. The transformed binary mask was multiplied with the normalized T_1_W image to skull strip the image. The resolution in the masked normalized image was doubled in all dimensions, and then the image was sharpened to further increase the intensity gradient at the pial surface. An intensity threshold was applied to the skull stripped image that was derived using an intensity histogram to calculate the threshold to remove CSF. A morphological algorithm to remove small objects was applied to remove dura mater. The new cerebrum mask was first eroded, then dilated to further remove dura mater. Following this, the cerebrum mask was down sampled back to the original image dimensions. One final threshold operation was applied to remove CSF that may have been added in during the dilation operation. This threshold intensity value was the same as was calculated earlier using the intensity histogram. All segmentations were manually inspected and corrected to remove any remaining dura mater or cerebellum using ITK-SNAP. Each subject’s group was blinded to the investigator until after the completion of all image processing.

The cerebrum segmentation was used to mask the locally normalized T_1_W created earlier. The masked image was used as the input to the FANTASM algorithm (Pham, 2001) in MIPAV, which segmented the image into two tissue classes: WM and GM. Following segmentation, labels for each tissue class were split back into left and right hemispheres for subsequent processing. A volume-preserving cortical depth model was used to generate the middle depth intracortical surface (Waehnert et al., 2014). Additionally, the ICBM-152 asymmetric atlas was processed equivalently to each subject, with automatic GM and WM segmentations that were manually inspected for accuracy, and a middle depth surface generated using a volume-preserving model. Each subject’s surface was registered to the middle depth surface generated from the ICBM-152 atlas using a multi-scale surface registration approach (Tardif et al., 2015). Each hemisphere was registered separately to improve speed and accuracy. Surfaces labeled with the T_1_W/T_2_W ratio intensity were created using CBS-Tools.

As B_1_ maps were not collected at time of imaging to correct inhomogeneities, an alternative approach was used to correct image intensities for analysis. This bias correction methodology is utilized in the preprocessing steps of the Human Connectome Project pipeline, and forms from the inverted contrast between the T_1_W and T_2_W images (Glasser et al., 2013). Bias field maps were thus calculated and applied to each subject’s images using FSL tools (Smith et al., 2004). Briefly, an initial estimate of the bias field forms from the square root of the product of the T_1_W and T_2_W images. The cortical mask that was generated in earlier processing steps of this study was applied to the bias map estimation and the resulting image was normalized to the mean value of the map, which now only contains values for inside the cerebrum. Smoothing was applied within the cortical mask, using a Gaussian smoothing kernel with a standard deviation of 5 mm. The resulting bias field was applied to both T_1_W and T_2_W images by a division of the image by the bias field. Finally, a corrected ratio image was generated by dividing the corrected T_1_W image by the corrected T_2_W image.

### Statistics

Cortical maps were generated using SurfStat^8^ in MATLAB, where signal intensity values were smoothed along the surface using a 6 mm Gaussian kernel. The Mars Atlas (Auzias et al., 2016) was used to parcellate the cortex into 82 ROIs for analysis with linear regressions computed using R^9^.

Signal intensity as a function of age was investigated for each ROI. As *ncvTest* in R reported non-equal error variance in the linear model, weighted regression was instead used for the analysis. The weight used was 1/standard deviation of the signal intensity for each ROI, which in effect applies a lower weighting to images with more artifacts such as noise. The regression equation took the form: *Signal ∼ Age* + *Study_site* + *Disease_group*, where *Study_site* was a factor with four levels corresponding to the location where the scans were taken. This was used to account for differences in bias fields and imaging methods at each site, and was validated by a lower Akaike criterion compared to using the scanner manufacturer. An age term was included because T_1_W/T_2_W signal intensity has been shown to vary with age in humans (Grydeland et al., 2013). Corrections for type-I errors were performed using the Holm–Bonferroni method (Ludbrook, 1998) on the resulting *p*-values calculated for each *Disease_group*, accounting for 82 ROIs.

## Results

### T_1_W/T_2_W Ratio Image Signal

**FIGURE 1:**
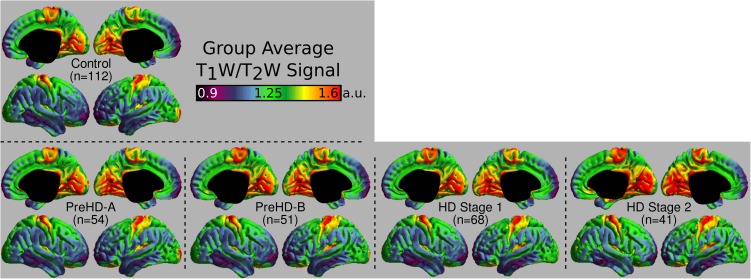
Middle depth T_1_W/T_2_W ratio signal for each study group. The effects of age and imaging site on the ratio signal were regressed out on a per-vertex basis for each subject before calculation of the average.

**FIGURE 2:**
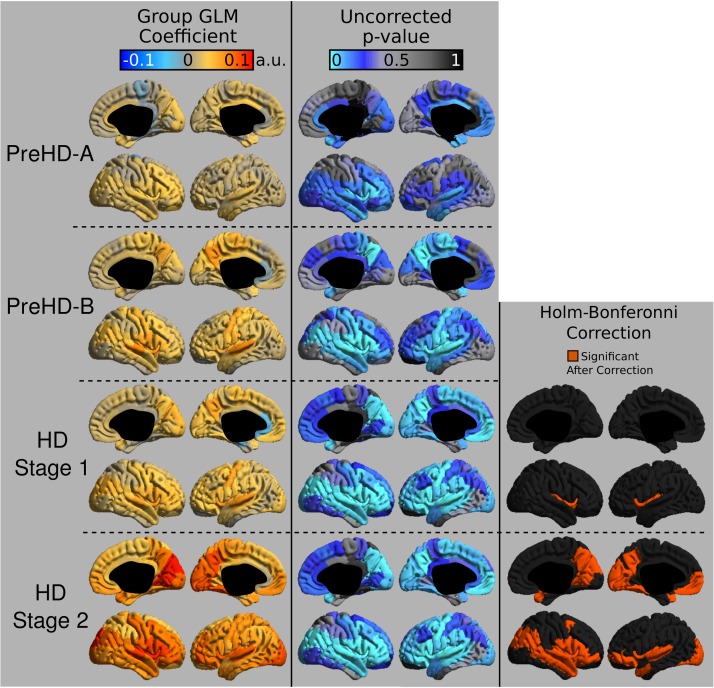
T_1_W/T_2_W ratio changes in HD. Coefficients from the intracortical signal analysis are mapped onto the cortex (left), illustrating the difference in ratio signal relative to controls with effects of age and imaging site regressed out of the signal. Uncorrected *p*-values generated for each group from the linear models are mapped onto the cortex for each region to visualize trends in changing intracortical signal (middle). Changes in intracortical signal that are significant following multiple comparison correction are found in HD Stage 1 and 2 (right).

The group signal maps calculated on per-vertex basis are displayed in Figure 1. This was computed to be the intercept of the regression equation that included Age and Study_site as regressors to the vertex signal from each subject. Following from this, differences from each HD group relative to the control group based on the T_1_W/T_2_W ratio image intensity are presented in Figure 2 as was conducted using the Mars Atlas cortical parcellation. The regression coefficients are presented for each group to illustrate the difference relative to the control population. This value represents the residual difference in ratio signal after age and study site are regressed out of the data. Though insignificant, there was a trend for a decrease in T_1_W/T_2_W ratio in the medial precentral gyrus and posterior cingulate cortex in the PreHD-A group. In other cortical regions, ratio intensity predominately demonstrated trends for increasing intensity relative to controls. This trend for increasing intracortical T_1_W/T_2_W intensity was similar for groups: PreHD-A, PreHD-B and HD Stage 1. The *p*-value maps (Figure 2, middle; listed in Supplementary Table 1) illustrate the spread of areas with increasing signal from the insula to other brain regions with disease advancement, however, most of these regions were statistically insignificant following multiple comparison correction. In Stage 2, the furthest progression of the disease included in this study, showed trends for increasing ratio signal across the cortex. Significant regions were found in the HD Stage 1 and 2 group relative to controls following multiple comparison correction, and are shown in the right panel of Figure 2. The ratio signal increased in a similar pattern bilaterally within the following significant regions: insula, ventrolateral frontal cortex, superior temporal gyrus, medial temporal gyral pole, and cuneus.

## Discussion

In this study, we used T_1_W/T_2_W ratio images to map changes in cortical composition across HD time points. The increase found in HD could be reflecting increases in myelin and/or iron amounts, as both of these are prominent features of cortical composition that affect the MR signal (Fukunaga et al., 2010). A MRI voxel reporting an increase in myelin could be the result of overall increases in myelin sheaths, or an increased density of myelin due to neuronal loss and/or shrinkage. Oligodendrocytes, the myelin producing cells in the central nervous system, are known to double in density in presymptomatic HD gene carriers in the tail of the caudate nucleus, while other cell densities are unchanged (Gómez-Tortosa et al., 2001). Neuronal densities have been shown to decrease while oligodendrocyte density increases after HD onset in the caudate (Myers et al., 1991). While these findings are in subcortical GM, increased glial density been reported in the GM of the cortex with HD onset as well (Passani et al., 1997; Rajkowska et al., 1998; Selemon et al., 2003). These histological findings suggest that the signal increase could be reporting both an increase in myelin levels as well as decreases in neuronal densities, resulting in higher myelin proportions.

Additionally, the increase in ratio signal could be reporting increased levels of cortical iron. It has previously been reported that iron loaded in the ferritin protein increases in subcortical GM in HD (Bartzokis et al., 1999, 2007), and found only after HD onset (Dumas et al., 2012b). Ferritin iron has a strong effect on decreasing T_2_W signal, but only a weak effect on increasing T_1_W signal (Vymazal et al., 1995), where both of these changes would lead to an increase in the ratio studied. While there are conflicting MRI reports of cortical iron accumulation (Rosas et al., 2012) and iron decreases (Sánchez-Castañeda et al., 2014), both studies agree that iron concentrations become further altered the longer the disease progresses, a finding replicated in this current study. As histology work has reported increases in glia in both subcortical and cortical GM, it is likely that the increase in subcortical iron levels could be present in the cortex as well. Disentangling iron and myelin contributions to signal can be difficult, since oligodendrocytes are iron enriched cells (Todorich et al., 2009), and combined MRI and histology work has shown that there is considerable overlap in iron and myelin distributions in the cortex (Stüber et al., 2014). However, it has been shown that only in later stages of HD, ferritin accumulates in the microglia of both the cortical and the striatal tissue (Simmons et al., 2007). Thus the increase in ratio signal could be separate from changes in myelin, and be reporting increased ferritin levels in cortical microglia. This separation is worth further investigation due to iron’s role in oxidative stress, metabolism, and iron-dependent enzyme production, which may contribute to symptomology.

Finally, the increased ratio signal could be due to changes in cytoarchitecture of the cortex, such that there is less bulk water content per imaging voxel. Histological studies have shown that neuronal densities decrease in HD (Passani et al., 1997; Rajkowska et al., 1998; Selemon et al., 2003). This decrease in neuronal density and increase in glial density could therefore decrease MR visible water as it has been highlighted in the mammalian central nervous system that neurons have a higher water percentage than glial cells (LoPachin et al., 1991). However, Eickhoff et al. (2005), previously reported that myelin content dominates the cortical signal over the underlying cytoarchitecture, such that it is unlikely that the change in signal in HD is due to changes in cytoarchitecture. To obtain a better understanding regarding the underlying pathology using neuroimaging, more specific MRI contrasts should be used, such as magnetization transfer for investigating myelin (Helms et al., 2008), quantitative susceptibility mapping for iron (Langkammer et al., 2010) and diffusion imaging for exploring neurite density (Calamante et al., 2017).

The regional variations in signal in this study differ from the pattern of cortical pathology in previous reports from cortical thickness studies (Rosas et al., 2002, 2008; Tabrizi et al., 2009). It was not necessarily expected that these two analysis techniques would present the same results as they are aimed at differing biological mechanisms. Indeed, it has been shown that tissue composition changes in HD independently of gross structural changes (Dumas et al., 2012b). We did, however, see a trend for an early decrease in signal in the motor regions of the medial cortex, which overlaps with early cortical thinning reported in this region (Rosas et al., 2002, 2008; Tabrizi et al., 2009). Many of the significant regions found in Stage 2 HD overlap with a previous statistical map depicting regions of cortical thinning correlating with verbal fluency scores in HD (Rosas et al., 2008). Our finding of the ventrolateral frontal cortex being predominately affected in Stage 2 HD may have structural significance given that this area projects to the caudate (Cummings, 1993), a structure that exhibits rapid degeneration in HD. Ventrolateral frontal cortex appears to be involved in working memory and contextual retrieval (Owen, 1997; Chapados and Petrides, 2015), which could be considered be among the executive functions HD patients experience deterioration in (Walker, 2007).

In this study, it was found that the earliest significant region with increased ratio signal following multiple-comparison correction was the insula. Insular atrophy in HD has previously been correlated with executive dysfunction (Peinemann et al., 2005). Microglia activation in the insula has been shown to be associated with neuronal loss in this area early in the disease (Tai et al., 2007). Additionally degeneration of the insula, as well as frontal-striatal circuits has been associated with apathy severity scores in HD (Martínez-Horta et al., 2018). Taken together, insular degeneration appears to be linked strongly with disease symptomology, and future work may look to use the MRI signal in the insula to track HD progression and symptom worsening.

A major limitation of this study is that motion may affect the quality of the MRI image. Due to the retrospective nature of the study, we were unable to control for patient motion. As HD is a movement disorder, it is possible that our results could be confounded by increased subject movement correlating with disease progression. However, care was taken with manual inspection of images during the segmentation step to only include images that appeared motion-free. Additionally, previous work in HD supports the time points of our findings, with late onset showing the greatest changes in tissue composition (Rosas et al., 2012).

An additional limitation is that there remains a residual bias field artifact in the ratio image after division, which could not be analytically corrected without B_1_ maps. Differences in radiofrequency coil geometries between different MRI vendors and sites can mean that the T_1_W and T_2_W images have intensity bias fields that differ between sites. The T_1_W/T_2_W ratio does well at removing much of the receive field bias (B_1_−), but incomplete canceling of transmit bias (B_1_+). While a correction method was applied to aid in removing some of the residual bias (Glasser et al., 2013), a proper analytical correction with a B_1_ map would be preferred for accuracy. This inter-site difference was also accounted for in the linear model, where a site term was included to account for inter-site differences in signal that arise from bias fields. Additionally, subjects were removed from analysis that exhibited an obvious gain difference in either the T_1_W or T_2_W image, which would scale the resulting ratio image. A T_1_W/T_2_W calibration algorithm was previously proposed which would scale the contrast in each image to muscle and eye intensity (Ganzetti et al., 2014). However, the calibration algorithm was deemed inappropriate to apply as changes in skeletal muscular structure have been shown in HD (Zielonka et al., 2014), such that calibrating to muscle in HD may also provide a confounding factor.

## Conclusion

In summary, it was found that HD progression induces widespread changes in cortical tissue composition, which was visible in a large number of subjects using standard anatomical images. It would be valuable to explore cortical pathology in HD using MRI methods that are more specific for features of cortical tissue composition, to better determine whether iron, myelin or both are responsible for this change in ratio signal. Additionally, revisiting this study with imaging optimized for intracortical contrast and MRI protocols that account for intensity biases between imaging sites, may provide increased sensitivity for detecting subtle cortical changes earlier in HD with MRI. With increased sensitivity, it would also be valuable to correlate changes in cortical composition with clinical and behavioral variables as well as other MRI metrics such as DTI. Furthermore, optimized imaging for cortical contrast may allow for detecting HD related changes in smaller cohorts. If intracortical signal mapping can track HD through more disease stages, it may have the potential to be used as a biomarker to mark disease progression in clinical trials.

## Ethics Statement

The study was approved by the local ethics committees from each imaging site, and written informed consent was obtained from each participant.

## Author Contributions

CR processed the images, performed the statistics, and drafted the manuscript. ST, RS, BL, RR, and AD conducted the original TRACK-HD study, provided the data for this analysis, and aided in manuscript preparation. NB aided in analysis and manuscript preparation.

## Conflict of Interest Statement

The authors declare that the research was conducted in the absence of any commercial or financial relationships that could be construed as a potential conflict of interest.

## Footnotes

1 https://www.mathworks.com

2 http://www.itksnap.org/

3 mipav.cit.nih.gov

4 www.nitrc.org/projects/jist/

5 www.nitrc.org/projects/toads-cruise

6 www.nitrc.org/projects/cbs-tools

7 http://nist.mni.mcgill.ca

8 http://www.math.mcgill.ca/keith/surfstat/

9 https://www.r-project.org

## Abbreviations

ACC: anterior cingulate cortex
CSF: cerebral spinal fluid
GM: gray matter
HD: Huntington’s Disease
*HTT*: huntingtin
MRI: magnetic resonance imaging
ROI: region of interest
T_1_W: T_1_-weighted
T_2_W: T_2_-weighted
WM: white matter.
