# Supplementary material for: Altered Intracortical T1-Weighted/T2-Weighted Ratio Signal in Huntington’s Disease
Source: Front Neurosci. 2018 Nov 5;12:805. doi: 10.3389/fnins.2018.00805 (PMC6230564; doi:10.3389/fnins.2018.00805)
Supplement: Supplementary file 1 [file Table_1.DOCX]

|  | **Left Hemisphere ROIs** | | | | **Right Hemisphere ROIs** | | | |
| --- | --- | --- | --- | --- | --- | --- | --- | --- |
| **ROI Names** | **PreHD-A** | **PreHD-B** | **HD Stage 1** | **HD Stage 2** | **PreHD-A** | **PreHD-B** | **HD Stage 1** | **HD Stage 2** |
| Caudal Medial Visual Cortex | 0.44 | 0.49 | 0.087 | **0.00031** | 0.20 | 0.507 | 0.063 | 0.0014 |
| Lateral Visual Cortex | 0.36 | 0.48 | 0.44 | 0.0011 | 0.29 | 0.497 | 0.28 | 0.0036 |
| Superior Visual Cortex | 0.69 | 0.30 | 0.29 | 0.014 | 0.18 | 0.042 | 0.011 | **0.00055** |
| Cuneus | 0.46 | 0.16 | 0.061 | **0.00084** | 0.17 | 0.200 | 0.018 | **2.73E-05** |
| Rostral Medial Visual Cortex | 0.27 | 0.28 | 0.37 | 0.055 | 0.75 | 0.344 | 0.22 | **0.00021** |
| Medial Inferior Temporal Cortex | 0.97 | 0.52 | 0.41 | 0.24 | 0.98 | 0.549 | 0.83 | 0.022 |
| Rostral Inferior Temporal Cortex | 0.08 | 0.66 | 0.70 | 0.0048 | 0.18 | 0.699 | 0.55 | 0.0025 |
| Caudal Middle Temporal Cortex | 0.20 | 0.16 | 0.039 | 0.0021 | 0.15 | 0.070 | 0.044 | **0.00051** |
| Caudal Superior Temporal Cortex | 0.11 | 0.0017 | 0.0011 | **0.00019** | 0.12 | 0.002 | 0.0041 | **0.00011** |
| Rostral Superior Temporal Cortex | 0.37 | 0.086 | 0.12 | 0.0088 | 0.20 | 0.047 | 0.033 | **0.00021** |
| Rostral Middle Temporal Cortex | 0.12 | 0.072 | 0.11 | **0.00051** | 0.077 | 0.098 | 0.043 | **9.75E-05** |
| Isthmus Cingulate Cortex | 0.44 | 0.044 | 0.13 | 0.38 | 0.61 | 0.829 | 0.35 | 0.042 |
| Ventral Inferior Parietal Cortex | 0.29 | 0.12 | 0.055 | 0.0056 | 0.19 | 0.042 | 0.024 | 0.0010 |
| Dorsal Inferior Parietal Cortex | 0.75 | 0.27 | 0.23 | 0.041 | 0.74 | 0.400 | 0.35 | 0.099 |
| Superior Parietal Cortex | 0.57 | 0.19 | 0.34 | 0.011 | 0.50 | 0.266 | 0.49 | 0.039 |
| Medial Superior Parietal Cortex | 0.38 | 0.066 | 0.11 | 0.0047 | 0.54 | 0.687 | 0.80 | 0.10 |
| Medial Parietal Cortex | 0.24 | 0.0032 | 0.006 | **0.00013** | 0.33 | 0.007 | 0.045 | **0.00065** |
| Posterior Cingulate Cortex | 0.14 | 0.088 | 0.25 | 0.10 | 0.97 | 0.289 | 0.83 | 0.061 |
| Ventral Somatosensory Cortex | 0.28 | 0.016 | 0.034 | 0.0021 | 0.14 | 0.009 | 0.017 | **0.00057** |
| Dorsolateral Somatosensory Cortex | 0.68 | 0.13 | 0.19 | 0.032 | 0.47 | 0.109 | 0.17 | 0.0028 |
| Dorsomedial Somatosensory Cortex | 0.66 | 0.11 | 0.29 | 0.0082 | 0.83 | 0.106 | 0.31 | 0.018 |
| Ventral Motor Cortex | 0.58 | 0.042 | 0.069 | 0.014 | 0.15 | 0.005 | 0.014 | **8.74E-05** |
| Dorsolateral Motor Cortex | 0.49 | 0.050 | 0.015 | 0.018 | 0.36 | 0.051 | 0.081 | 0.0086 |
| Dorsomedial Motor Cortex | 0.31 | 0.043 | 0.057 | 0.031 | 0.69 | 0.200 | 0.59 | 0.21 |
| Rostral Ventral Premotor Cortex | 0.30 | 0.11 | 0.0081 | 0.0013 | 0.065 | 0.009 | 0.0037 | **1.62E-05** |
| Dorsolateral Premotor Cortex | 0.48 | 0.18 | 0.047 | 0.015 | 0.17 | 0.110 | 0.039 | **0.00072** |
| Dorsomedial Premotor Cortex | 0.21 | 0.25 | 0.064 | 0.087 | 0.76 | 0.320 | 0.26 | 0.24 |
| Caudal Dorsolateral Prefrontal Cortex | 0.68 | 0.30 | 0.23 | 0.0098 | 0.17 | 0.101 | 0.10 | 0.0024 |
| Caudal Dorsomedial Prefrontal Cortex | 0.53 | 0.68 | 0.091 | 0.075 | 0.53 | 0.632 | 0.18 | 0.091 |
| Mid Cingulate Cortex | 0.08 | 0.15 | 0.33 | 0.18 | 0.29 | 0.239 | 0.58 | 0.20 |
| Rostral Ventrolateral Prefrontal Cortex | 0.29 | 0.077 | 0.023 | 0.0013 | 0.05 | 0.023 | 0.0047 | **0.0000132** |
| Rostral Dorsolateral Inferior Prefrontal Cortex | 0.34 | 0.23 | 0.090 | 0.0017 | 0.11 | 0.200 | 0.020 | 0.0015 |
| Rostral Dorsolateral Superior Prefrontal Cortex | 0.58 | 0.21 | 0.062 | 0.0031 | 0.17 | 0.230 | 0.095 | 0.0033 |
| Rostral Dorsal Prefrontal Cortex | 0.33 | 0.62 | 0.30 | 0.17 | 0.34 | 0.465 | 0.17 | 0.27 |
| Rostral Medial Prefrontal Cortex | 0.13 | 0.32 | 0.058 | 0.0099 | 0.42 | 0.274 | 0.27 | 0.025 |
| Ventrolateral Orbito Frontal Cortex | 0.64 | 0.10 | 0.021 | **0.00021** | 0.29 | 0.091 | 0.031 | **0.00014** |
| Ventral Orbito Frontal Cortex | 0.28 | 0.19 | 0.066 | **0.00035** | 0.13 | 0.106 | 0.038 | **0.00047** |
| Ventromedial Orbito Frontal Cortex | 0.19 | 0.93 | 0.87 | 0.042 | 0.10 | 0.384 | 0.26 | 0.071 |
| Ventromedial Prefrontal Cortex | 0.13 | 0.21 | 0.020 | **0.00049** | 0.64 | 0.446 | 0.18 | 0.0020 |
| Anterior Cingulate Cortex | 0.88 | 0.77 | 0.18 | 0.56 | 0.14 | 0.335 | 0.25 | 0.070 |
| Insular Cortex | 0.28 | 0.0093 | **0.00040** | **7.55E-05** | 0.051 | 0.00066 | **0.00025** | **2.49E-06** |

**Supplementary Table 1:** *p*-values for the group coefficient in the general linear model. The *p*-value tests the difference relative to controls with age and study site effects regressed out from the data. Bold values remained significant following Bonferonni-Holm correction.
